# Performance of metagenomic next-generation sequencing in bronchoalveolar lavage fluid for pathogen detection in patients with acute exacerbations of bronchiectasis

**DOI:** 10.1097/MD.0000000000045606

**Published:** 2025-11-07

**Authors:** Qidong Zhuang, Ruyi Xu, Xinying Sun, Xiaodan Pan, Lihong Wan, Sha Li, Hui Chen, Xuechan Yu, Lin Zheng, Yiming Yu, Zaichun Deng, Xiaoxuan Zheng, Zhongbo Chen

**Affiliations:** a Department of General Practice, People’s Hospital of Zhenhai, Ningbo, Zhejiang Province, China; b Department of Critical Care Medicine, Ningbo Traditional Chinese Medicine Hospital, Ningbo, China; c Department of Respiratory and Critical Care Medicine, Key Laboratory of Respiratory Disease of Ningbo, The First Affiliated Hospital of Ningbo University, Ningbo, China; d Department of Respiratory Medicine, Unit 94969 of the Chinese People’s Liberation Army, Shanghai, China; e Respiratory and Critical Care Medicine, The Second Affiliated Hospital of Zhejiang University School of Medicine, Hangzhou, China; f Department of Microbiology, The First Affiliated Hospital of Ningbo University, Ningbo, China; g Department of Respiratory Endoscopy, Shanghai Chest Hospital, Shanghai Jiao Tong University School of Medicine, Shanghai, China.

**Keywords:** acute exacerbations, bronchiectasis, bronchoalveolar lavage fluid, metagenomic next-generation sequencing, pathogen

## Abstract

This study aimed to evaluate the diagnostic value of metagenomic next-generation sequencing (mNGS) of bronchoalveolar lavage fluid (BALF) in detecting pathogens among bronchiectasis patients with acute exacerbations. A retrospective analysis was conducted on 89 bronchiectasis patients who were treated for acute exacerbations at the First Affiliated Hospital of Ningbo University from April 1, 2021, to September 30, 2023. Among the 89 patients, 88 were diagnosed with pulmonary infection, of which 15.9% (14/88) were cases of mixed infections. The sensitivity of BALF-mNGS for detecting pathogens in bronchiectasis patients during acute exacerbations was significantly higher than that of BALF culture (93.2% vs 28.4%; *P* < .001). All cases of mixed infection were fully identified by BALF-mNGS. The most common pathogens in patients with bronchiectasis were *Pseudomonas aeruginosa*, nontuberculous mycobacteria, *Haemophilus influenzae*, and *Aspergillus*. In conclusion, compared with the traditional microbial culture method, BALF-mNGS significantly improves the accuracy of diagnosis for detecting pathogens associated with bronchiectasis infections.

## 1. Introduction

Bronchiectasis is a common progressive chronic airway disease, characterized by airway remodeling, colonization, and recurrent infectious exacerbation caused by microbial expansion.^[1,2]^ In the United States, some scholars have reported that the prevalence rate was 701 per 1,00,000 individuals.^[3]^ Similar rates have been reported in the United Kingdom, Spain, and Germany.^[4–6]^ In China, the overall prevalence of bronchiectasis in people aged 40 years or older was estimated at 1.2% and was trending upward with aging of the population.^[7]^ This substantial patient population has raised awareness of the recognition of an urgent need for effective therapeutic interventions for the management of bronchiectasis, particularly during acute exacerbations.

Previous studies have demonstrated that viruses, fungi, and bacteria are more prevalent in bronchiectasis patients during acute exacerbations.^[8,9]^ However, the diagnostic efficiency of conventional methods is constrained by the high diversity of respiratory tract infection pathogens, as well as the presence of commensal microbiota and pathobionts in the respiratory tract.^[10]^ For instance, microbial culture is time-consuming and incapable of detecting viruses and parasites,^[11]^ while antigen/antibody assays may exhibit poor sensitivity.^[12]^ Although conventional polymerase chain reaction (PCR) based nucleic acid detection has high sensitivity and specificity, it is limited in detecting a broad range of microorganisms and may fail to identify the pathogen responsible for the infection.^[13]^ Therefore, urgently needed are pathogen detection and identification methods for bronchiectasis with higher diagnostic efficiency to overcome the limitations in turnaround time, sensitivity, specificity, and diagnostic spectrum.

Metagenomic next-generation sequencing (mNGS) has been proposed as a valuable tool for pathogen identification in infectious diseases due to its comprehensiveness, speed, high sensitivity, and specificity.^[14]^ The clinical utility of mNGS has been confirmed in diagnosing invasive pulmonary aspergillosis,^[15]^ nontuberculous mycobacteria (NTM) pulmonary disease.^[16]^ Nonetheless, the performance of bronchoalveolar lavage fluid (BALF) mNGS for pathogen detection in patients with bronchiectasis remains underexplored. This study aims to evaluate the value of BALF-mNGS in identifying pathogens in patients with acute exacerbations of bronchiectasis.

## 2. Materials and methods

### 2.1. Study patients

This study was approved by the Ethics Committee of the First Affiliated Hospital of Ningbo University (Ningbo, China). Retrospective analysis of bronchiectasis patients with acute exacerbations who underwent bronchoscopy and comprehensive pathogen testing via mNGS of BALF from April 1, 2021, to September 30, 2023, at the First Affiliated Hospital of Ningbo University. Excluded: those who could not provide informed consent; those who were under 18 years of age; and (3) those with incomplete clinical data. A total of 89 patients were finally included in the analysis.

### 2.2. Bronchoalveolar lavage, DNA extraction, and sequencing

Under general anesthesia, bronchoscopy was performed by a professional physician and bronchoalveolar lavage was performed 3 times (20 mL each time). The lavage area was determined based on the location of the lesion shown by chest computed tomography. In cases of diffuse disease in both lungs, lavage was performed in the middle lobe of the right lung.^[17]^ Two tubes of 5 mL BALF were immediately sealed and stored for subsequent mNGS detection. About 1.2 mL phosphate buffer solution (10 mM, pH 7.2–7.4) was added to the specimen tube, and the mixture was shaken and mixed for 10 minutes in a vortex shaker. The liquid from the 1.2 mL specimen tube was aspirated into a 2 mL shaker tube. The shaker tube was placed in a biological sample homogenizer, homogenized for 9 minutes. During this process, the samples were ground, lysed, and homogenized through high-efficiency 3-dimensional high-speed collisions, which effectively shattered the cells. The shaker tube was removed, transferred to a centrifuge and centrifuged at 12,000 rpm for 3 minutes. Subsequently, 400 μL of the supernatant was aspirated into the small genomic nucleic acid extraction or purification kit, which was then proceed in the NGS master automatic library construction instrument for nucleic acid extraction and library preparation. Double-stranded DNA libraries were then converted into single-stranded circular DNA using DNA degradation and cyclizing. Finally, high-throughput sequencing was performed on Illumina sequencing platform.^[18,19]^

### 2.3. Microbial culture

The microbial culture procedure was performed by clinical laboratory professionals in strict compliance with quality control criteria for BALF samples. Samples eligible for culturing were selected based on the following criteria: absence of significant blood contamination with erythrocyte counts below 10% and a general epithelial cell content <3%. The procedures for BALF bacterial and fungal cultures were conducted as follows: For bacterial culture, BALF samples were first vortexed for 60 seconds. Subsequently, 10 μL aliquots were inoculated onto a blood agar plate and a chocolate agar plate, respectively. The inoculated plates were incubated at 35°C in an atmosphere containing 6% carbon dioxide for a standard incubation period of 48 hours. For fungal culture, BALF samples were centrifuged, and the sediment was subsequently collected and mixed before being inoculated onto 2 Sabouraud’s agar plates. The inoculated plates were then incubated at 25 and 35°C for 7 days, respectively. Mass spectrometry was employed to identify the cultured microbial organisms.

### 2.4. Final diagnosis and evaluation of diagnostic performance for mNGS and culture

Due to the lack of currently recognized diagnostic guidelines or consensus for etiological diagnosis based solely on mNGS test results, in this study, we referred to previous studies and adopted a comprehensive approach to reach a final diagnosis.^[20,21]^ In addition to the information provided in the mNGS report regarding the detection of infectious pathogens, we also took into account symptoms and signs associated with the infection. The results of other routine microbiological tests (such as bacterial and fungal smear cultures, real-time PCR, serum indirect immunofluorescent antibodies, galactomannan antigen, (1,3)-β-d-glucan), laboratory tests (C-reactive protein, procalcitonin, white blood cell count, neutrophil count) and radiologic findings were evaluated. Besides, treatment regimens and outcomes were incorporated into the assessment. For patients with complex conditions, their final diagnoses were established through a collaborative evaluation by multiple experienced experts.

The interpretation of BALF-mNGS test results and the final diagnosis can be summarized as follows: A true positive is a pathogen detection matching the final diagnosis; a false positive is a detected pathogen inconsistent with the diagnosis. A true negative indicates no pathogens detected in noninfectious cases, while a false negative is a missed causative pathogen.

### 2.5. Statistical analysis

A normality test was performed on metric data, with normally distributed data represented as mean ± standard deviation, and skewed data was presented as median (interquartile range). The comparison of count data between 2 groups was conducted using the chi-square test, while the comparison of metric data between 2 groups was performed using analysis of variance (for normally distributed data) or nonparametric tests (for non-normally distributed data). A significance level of *P *< .05 was used to define statistical significance for all 2-tailed tests.

## 3. Results

### 3.1. Patient characteristics

The demographic characteristics of the patients recruited for this study are summarized in Table 1. There were 42 males and 47 females, with a median age of 66 years. The predominant clinical symptoms included cough (87.6%), expectoration (82.0%), fever (28.1%), dyspnea (23.6%), chest tightness (18.0%), hemoptysis (16.9%), and chest pain (5.6%).

**Table 1:** Patient baseline characteristics.

Characteristics	All patients (N = 89)
Gender, N (%)	
Male	42 (47.19)
Female	47 (52.80)
Age (yr)	66.00 (57.50, 72.00)
Underlying disease, N (%)	
Chronic obstructive pulmonary disease	8 (9.0)
Asthma	9 (10.1)
Lung cancer	4 (4.9)
Hematologic neoplasms	7 (7.9)
Received chemotherapy or radiation	6 (6.7)
Diabetes	6 (6.7)
Steroids or immunosuppressive drugs	7 (7.9)
Primary clinical symptoms, N (%)	
Fever	25 (28.1)
Cough	78 (87.6)
Expectoration	73 (82.0)
Hemoptysis	15 (16.9)
Polypnea	21 (23.6)
Chest tightness	16 (18.0)
Chest pain	5 (5.6)
Laboratory examination	
White blood cell (×10^9^/L)	6.40 (4.85, 8.20)
Neutrophils count (×10^9^/L)	4.40 (3.07, 6.45)
Lymphocytes count (×10^9^/L)	1.40 (0.90, 1.80)
C-reactive protein (mg/L)	8.77 (1.92, 30.01)

### 3.2. Comparison of diagnostic performance of BALF culture and mNGS

BALF specimens from 89 patients were subjected to both mNGS testing and pathogen culture for comparison with the final diagnosis. The results demonstrated that BALF-mNGS achieved a sensitivity of 93.2%, specificity of 100%, positive predictive value of 100%, and negative predictive value of 14.3% in the identification of pathogens in patients with bronchiectasis. The sensitivity of BALF culture for diagnosing infectious diseases in patients with bronchiectasis was 28.4%, with a specificity of 100%, a positive predictive value of 100% and a negative predictive value of 1.6%. The sensitivity of mNGS was significantly higher than that of the culture method (93.2% vs 28.4%; *P* < .001; Table 2).

**Table 2:** Diagnostic performance of mNGS and culture tests for infections in patients with bronchiectasis.

	Sensitivity (95% CI)	Specificity (95% CI)	Positive predictive value (95% CI)	Negative predictive value (95% CI)
mNGS	93.2% (93.02–99.94%)	100% (5.46–100%)	100% (94.42–100%)	14.3% (0.75–57.99%)
Culture	28.4% (19.55–39.18%)	100% (5.46–100%)	100% (83.42–100%)	1.6% (0.08–9.54%)

CI = confidence interval, mNGS = metagenomic next-generation sequencing.

### 3.3. Types of infection in patients with bronchiectasis

**Figure 1.**
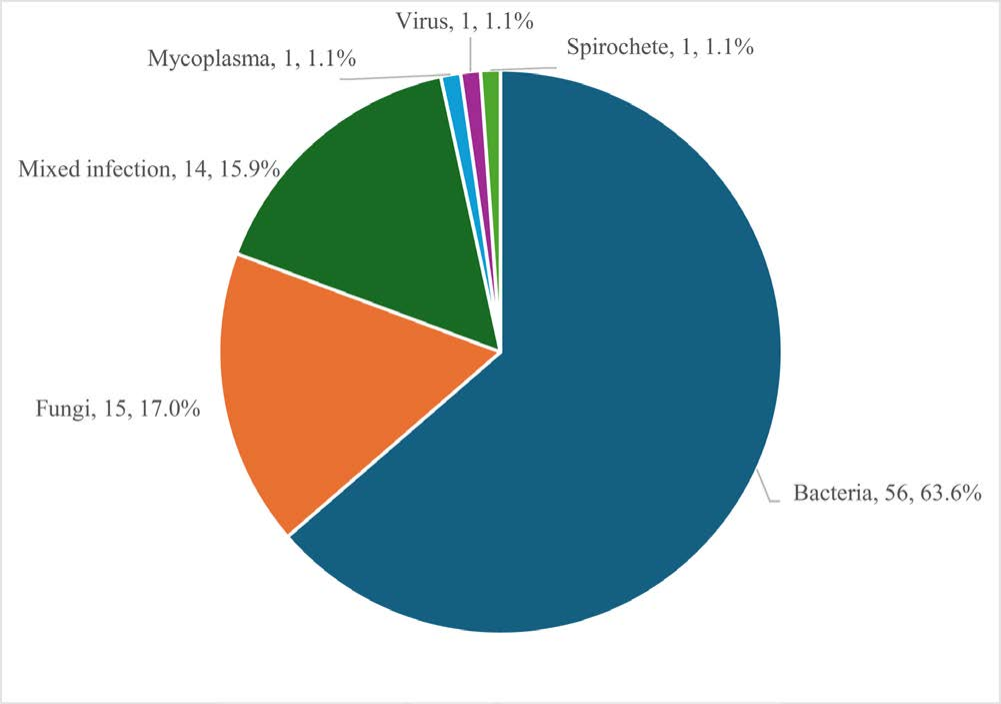
Types of infections in patients with bronchiectasis. Among 88 infected patients, 56 (63.6%) had a single bacterial infection, 15 (17.0%) had a single fungal infection, 1 (1.1%) had a viral infection, 1 (1.1%) had a spirochete infection, 1 (1.1%) had a mycoplasma infection, and 14 (15.91%) cases had a mixed infection.

According to the final diagnosis, 88 of the 89 patients were diagnosed with acute exacerbation of bronchiectasis accompanied by infection, including 74 patients with single-pathogen infections and 14 patients with mixed infections. Among these single-pathogen infected patients, 56 (63.6%) had a single bacterial infection, 15 (17.0%) had a single fungal infection, 1 (1.1%) had a viral infection, 1 (1.1%) had a spirochete infection and 1 (1.1%) had a mycoplasma infection (Fig. 1). Fourteen (15.9%) cases were identified as mixed infections, including 11 (12.5%) with co-infection of bacteria and fungi, and 3 (3.4%) with 2 or more bacterial infections. The pathogens responsible for these mixed infections were all detected by mNGS.

### 3.4. Pathogens detected in single-pathogen infected patients with bronchiectasis by mNGS and culture

**Figure 2.**
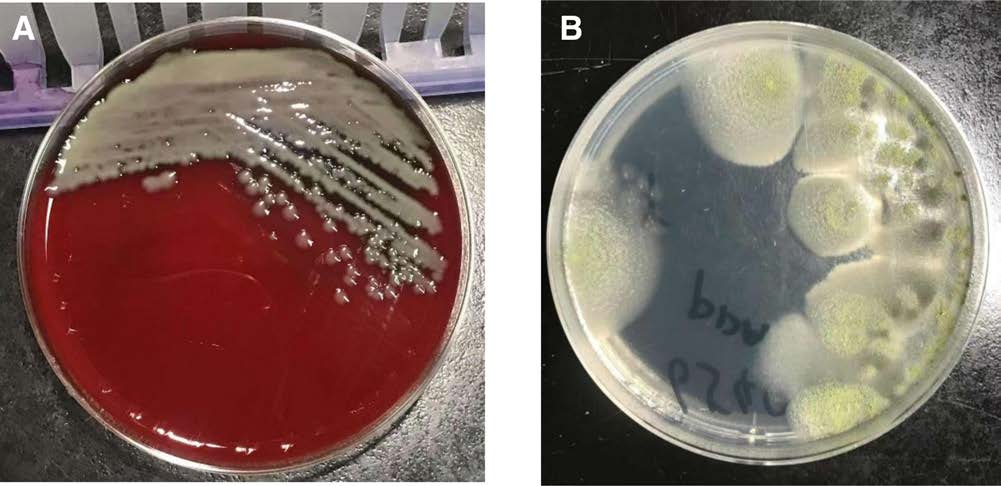
(A) *Pseudomonas aeruginosa* was cultured on the blood agar plate, and the BALF sample was obtained from a patient infected with a single-pathogen. (B) *Aspergillus fumigatus* was cultured on the Sabouraud’s agar plate, and the specimen was collected from a patient with a mixed infection. BALF = bronchoalveolar lavage fluid.

**Figure 3.**
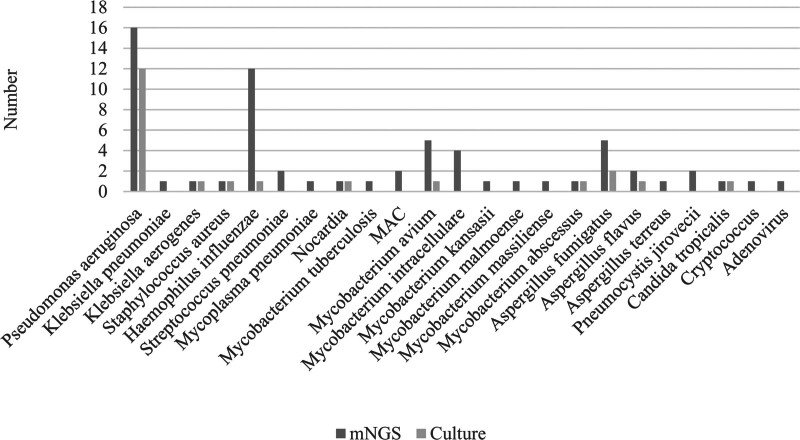
Major pathogens detected by culture and mNGS methods. A total of 10 pathogens, predominantly bacteria and fungi, were detected in BALF cultures of patients with bronchiectasis infected by a single-pathogen. A total of 82 patients with bronchiectasis complicated by pulmonary infection were diagnosed by mNGS. BALF = bronchoalveolar lavage fluid, MAC = *Mycobacterium avium*-intracellular complex, mNGS = metagenomic next-generation sequencing.

A total of 10 pathogens, predominantly consisting of bacteria and fungi, were identified in BALF cultures of patients with bronchiectasis infected by a single-pathogen. *Pseudomonas aeruginosa* was the most common causative agent, isolated from 12 BALF specimens (Fig. 2A). Other pathogens included *Klebsiella aerogenes* (n = 1), *Streptococcus pneumoniae* (n = 1), *Haemophilus influenzae* (n = 1), *Nocardia* (n = 1), *Mycobacterium avium* (n = 1), *M abscessus* (n = 1), *Aspergillus fumigatus* (n = 2), *Aspergillus flavus* (n = 1), and *Candida tropicalis* (n = 1). A total of 82 patients with bronchiectasis complicated by pulmonary infection were diagnosed using mNGS. And 25 species of pathogens were identified in patients with single-pathogen infections, including bacteria (n = 15), fungi (n = 7), virus (n = 1), mycoplasma (n = 1), spirochete (n = 1). The main pathogens were *P aeruginosa* (n = 16), NTM (n = 15), *H influenzae* (n = 12), *Aspergillus* (n = 8), and *S pneumoniae* (n = 2; Fig. 3). NTM included *M avium* (n = 5), *Mycobacterium intracellulare* (n = 4), *M avium-intracellular complex* (n = 2), *Mycobacterium kansasii* (n = 1), *Mycobacterium malmoense* (n = 1), *Mycobacterium massiliense* (n = 1) and *M abscessus* (n = 1). *Aspergillus* species included *A flavus* (n = 5), *Aspergillus terreus* (n = 2), and *A fumigatus* (n = 1).

### 3.5. Pathogens detected in mixed infected patients with bronchiectasis by mNGS and culture

Among 89 patients with bronchiectasis, 14 cases of mixed infections were identified, all of which were fully confirmed by mNGS. Of these 14 cases, BALF cultures were negative in 4 cases, while partial pathogenic agents were detected in the remaining 10 cases (Table 3). The most common pathogens in mixed infections were *P aeruginosa, Klebsiella pneumoniae*, NTM, and *Aspergillus* (Fig. 2B).

**Table 3:** Detailed pathogens information of samples with mixed infection.

ID	Definitive diagnosis	Bacterial and fungal culture	Metagenomic next-generation sequencing
5	*Talaromyces marneffei* *Aspergillus fumigatus* *Mycobacterium intracellulare*	Negative	*Talaromyces marneffei* *Aspergillus fumigatus* *Mycobacterium intracellulare*
8	*Pseudomonas aeruginosa* *Mycobacterium avium*	*Pseudomonas aeruginosa*	*Pseudomonas aeruginosa* *Mycobacterium avium*
15	*Klebsiella pneumoniae* Cryptococcus	*Klebsiella pneumoniae*	*Klebsiella pneumoniae* Cryptococcus
21	*Pseudomonas aeruginosa* *Klebsiella pneumoniae* *Staphylococcus aureus*	*Pseudomonas aeruginosa* *Klebsiella pneumoniae*	*Pseudomonas aeruginosa* *Klebsiella pneumoniae* *Staphylococcus aureus*
26	*Klebsiella pneumoniae* *Aspergillus fumigatus*	*Aspergillus*	*Klebsiella pneumoniae* *Aspergillus fumigatus*
59	*Mycobacterium avium* *Aspergillus fumigatus*	Negative	*Mycobacterium avium* *Aspergillus fumigatus*
63	*Klebsiella pneumoniae* *Aspergillus fumigatus*	*Aspergillus fumigatus*	*Klebsiella pneumoniae* *Aspergillus fumigatus*
65	*Mycobacterium avium* *Aspergillus flavus*	*Aspergillus flavus*	*Mycobacterium avium* *Aspergillus flavus*
67	*Pseudomonas aeruginosa* *Haemophilus influenzae*	*Pseudomonas aeruginosa*	*Pseudomonas aeruginosa* *Haemophilus influenzae*
76	*Pseudomonas aeruginosa* *Mycobacterium avium* *Aspergillus terreus*	*Aspergillus*	*Pseudomonas aeruginosa* *Mycobacterium avium* *Aspergillus terreus*
78	*Klebsiella aerogenes* *Pneumocystis jirovecii*	Negative	*Klebsiella aerogenes* *Pneumocystis jirovecii*
79	*Klebsiella pneumoniae* *Schizophyllum commune*	*Klebsiella pneumoniae*	*Klebsiella pneumoniae* *Schizophyllum commune*
84	*Mycobacterium abscessus* *Pneumocystis jirovecii*	Negative	*Mycobacterium abscessus* *Pneumocystis jirovecii*
87	*Pseudomonas aeruginosa* *Aspergillus flavus*	*Pseudomonas aeruginosa*	*Pseudomonas aeruginosa* *Aspergillus flavus*

### 3.6. Comparison of inflammatory markers and symptoms in patients with different types of single-pathogen infection

Among the patients with single-pathogen infection, the distribution was as follows: 15 cases of fungal infections, 15 cases of NTM infections, and 40 patients of common bacterial infections (excluding 1 case of tuberculosis). Significant differences were observed in white blood cell count (*P* = .031) and neutrophil count (*P* = .028) among patients with different types of infection. Bonferroni multiple means comparison showed that white blood cell count and neutrophil count in patients with NTM infection were significantly lower than those in patients with common bacterial infection. There were no significant differences in symptoms among patients with different infection types (Table 4).

**Table 4:** In patients with single infection, the inflammatory indexes and symptoms of common bacterial infection, fungal infection, and NTM infection were compared.

	Fungi (N = 15)	Common bacterial (N = 40)	NTM (N = 15)	*P*-value
Inflammatory indicators				
White blood cell (×10^9^/L)	6.94 ± 2.18	6.55 (5.43, 8.70)	5.26 (4.60, 6.30)	.031*
Neutrophils count (×10^9^/L)	4.21 ± 2.15	4.45 (3.27, 6.58)	3.10 (2.80, 4.40)	.028*
C-reactive protein (mg/L)	17.65 (2.10, 70.5)	9.53 (2.05, 30.25)	4.30 (1.87, 22.45)	.366
Symptoms				
Fever	7 (46.7%)	8 (20.0%)	2 (13.3%)	.074
Cough	14 (93.3%)	37 (92.5%)	12 (80.0%)	.430
Expectoration	12 (80.0%)	34 (85.0%)	12 (80.0%)	.753
Hemoptysis	4 (26.7%)	7 (17.5%)	2 (13.3%)	.700
Polypnea	6 (40.0%)	10 (25.0%)	2 (13.3%)	.251
Chest tightness	4 (26.7%)	7 (17.5%)	2 (13.3%)	.700
Chest pain	1 (6.7%)	3 (7.5%)	0 (0.0%)	.697

NTM = nontuberculous mycobacteria.

* *P*< .05 was considered statistically significant.

## 4. Discussion

The tendency to experience exacerbations is a defining characteristic of bronchiectasis. Antibiotic use is generally recommended during the exacerbation period.^[22]^ Therefore, the timely and effective identification of microbial infection types in patients with acute bronchiectasis exacerbations, along with the use of targeted therapeutic interventions, is crucial. Although it is the most frequently utilized sample for routine clinical pathogen identification in bronchiectasis, sputum remains a suboptimal source due to its highly variable volume and contamination with oral and upper airway contents. Hence, we collected BALF samples to identify pathogens in the lower airways of bronchiectasis patients using mNGS, a technique that has been widely adopted for the rapid and accurate detection of pathogens in individuals with lower respiratory tract infections.^[23,24]^ In our retrospective analysis, the pathogen detection sensitivity of BALF-mNGS (93.2%) was significantly higher than that of conventional BALF culture (28.4%; *P* < .001). Moreover, BALF-mNGS demonstrated good specificity, PPV, and Youden index, indicating that compared with culture, mNGS is a more suitable method for detecting pathogens in the BALF of patients with acute bronchiectasis exacerbations. Chinese scholars have reported a similar conclusion based on the finding that, among 77 bronchiectasis patients with negative sputum culture, 66 had positive results from BALF-mNGS.^[8]^ That study also indicated that patients with acute exacerbations of bronchiectasis were more likely to experience mixed infections in the lower respiratory tract. In our research, 15.9% (14/88) of patients were diagnosed with mixed infections based on the results of BALF-mNGS. Among these patients, BALF cultures were negative in 4 cases, while partial pathogens were detected in 10 cases. This finding is attributed to the superior diagnostic performance of mNGS for various pathogens compared to conventional cultures,^[25]^ suggesting that mNGS can also serve as a valuable tool for identifying mixed infections in patients with acute exacerbations of bronchiectasis. In addition, it has been confirmed that mNGS is less likely to be influenced by prior antibiotic exposure.^[14]^

Acute exacerbations of bronchiectasis can be caused by a wide variety of pathogens, posing significant challenges for both microbiological diagnosis and clinical management.^[1]^ It is crucial to understand the microbial characteristics in the lower respiratory tract of patients experiencing acute exacerbations of bronchiectasis in order to guide clinical treatment effectively. Bacterial infections are predominantly implicated as the primary etiology in most cases of bronchiectasis exacerbation, particularly chronic infection with *P aeruginosa*, which serves as a key indicator of disease severity and exacerbation frequency.^[26,27]^ In our study, using mNGS technology, *P aeruginosa* (22/88) were the most common species isolated in patients with acute exacerbations of bronchiectasis, which was consistent with previous reports.^[25,28]^ In many countries, NTM infections are frequently observed in patients with bronchiectasis and may potentially serve as a causative factor in the pathogenesis of bronchiectasis.^[29,30]^ A study performed by American scholars found that nearly 30% of patients with bronchiectasis had NTM infections.^[31]^ However, another study based on the Bronchiectasis Research Registry database in the United States showed that up to 63% of patients with non-cystic-fibrosis bronchiectasis had a history of NTM disease or NTM isolation.^[32]^ In our cohort of patients with bronchiectasis and infection, the positivity rate of NTM detection was 23.9% (21/88), which was higher than that reported in another Chinese study.^[7]^ In addition, fungal pathogens, particularly *Aspergillus* and *Candida* species are frequently detected in patients with bronchiectasis, which can act as a pathogen or an allergen.^[33]^ Recently, *Aspergillus* has been increasingly recognized as a significant cause of frequent exacerbations in patients with bronchiectasis, which is associated with faster deterioration of lung function.^[8,34]^ In our study, *Aspergillus* was most common fungal pathogen. Among these patients, nearly half were diagnosed with mixed infections. Furthermore, viral infection can also trigger exacerbations in bronchiectasis. In a previous study conducted with 119 patients in China, investigators reported that viral infections detected by PCR assay occurred more frequently during exacerbation periods compared to steady-state bronchiectasis.^[35]^ In that cohort, *coronaviruses, rhinovirus*, and *influenza viruses* were the most commonly identified viral pathogens. However, only 1 patient was diagnosed with *adenovirus* infection in our research.

Our study further explored the association between the detection results of different pathogen subtypes and the symptoms and inflammatory indexes of bronchiectasis patients. Although symptoms were nonspecific in bronchiectasis patients with NTM, cough, and expectoration remained the main clinical manifestations, consistent with prior study.^[36]^ Notably, in NTM patients, white blood cell count and neutrophil count were significantly lower compared to those in patients with common bacterial infection (Table 4). This finding may assist clinicians in selecting appropriate antibiotics for initiating empirical anti-infective therapy.

This research has several limitations. First, it is a single-center study with a relatively small sample size, which may limit its generalizability to a broader population. Second, only DNA sequencing was performed, allowing for the detection of bacteria, fungi, and DNA viruses, but not RNA viruses. Third, the interpretation of mNGS results currently lacks a universally accepted standard, requiring physicians to integrate patients’ medical histories, clinical presentations, other laboratory test results, and imaging findings to make a final clinical diagnosis. This process often involves subjective judgment. Finally, BALF specimens were not obtained from healthy individuals because performing bronchoscopy on healthy persons would be unethical; thus, comparing airway microbiota profiles between healthy individuals and bronchiectasis patients is not feasible.

## 5. Conclusion

Compared with traditional BALF microbial culture, BALF-mNGS exhibits high sensitivity and the ability to detect a broad spectrum of pathogens. It also demonstrates advantages in detecting special pathogens, enabling rapid diagnosis and guiding precise treatment. Additionally, it can identify mixed infections and reduce the likelihood of missed or incorrect diagnoses.

## Acknowledgments

We appreciate all participants who took part in our study.

## Author contributions

**Investigation:** Qidong Zhuang, Ruyi Xu, Yiming Yu, Zaichun Deng.

**Conceptualization:** Ruyi Xu, Zhongbo Chen.

**Data curation:** Xiaodan Pan.

**Formal analysis:** Ruyi Xu, Xuechan Yu, Zaichun Deng, Zhongbo Chen.

**Methodology:** Ruyi Xu, Xinying Sun, Zhongbo Chen.

**Project administration:** Zaichun Deng, Zhongbo Chen.

**Supervision:** Zaichun Deng, Zhongbo Chen.

**Resources:** Zhongbo Chen.

**Visualization:** Ruyi Xu, Zhongbo Chen.

**Validation:** Sha Li, Hui Chen, Lin Zheng.

**Writing – original draft:** Qidong Zhuang, Ruyi Xu, Xinying Sun, Hui Chen.

**Writing – review & editing:** Qidong Zhuang, Ruyi Xu, Lihong Wan, Zaichun Deng, Xiaoxuan Zheng, Zhongbo Chen.

## References

[1] O’Donnell AE. Bronchiectasis - a clinical review. N Engl J Med. 2022;387:533–45. 35947710 10.1056/NEJMra2202819

[2] Metersky ML Barker AF. The pathogenesis of bronchiectasis. Clin Chest Med. 2022;43:35–46. 35236559 10.1016/j.ccm.2021.11.003

[3] Henkle E Chan B Curtis JR Aksamit TR Daley CL Winthrop KL. Characteristics and health-care utilization history of patients with bronchiectasis in US medicare enrollees with prescription drug plans, 2006 to 2014. Chest. 2018;154:1311–20. 30055168 10.1016/j.chest.2018.07.014

[4] Quint JK Millett ER Joshi M. Changes in the incidence, prevalence and mortality of bronchiectasis in the UK from 2004 to 2013: a population-based cohort study. Eur Respir J. 2016;47:186–93. 26541539 10.1183/13993003.01033-2015 PMC4982534

[5] Monteagudo M Rodríguez-Blanco T Barrecheguren M Simonet P Miravitlles M. Prevalence and incidence of bronchiectasis in Catalonia, Spain: A population-based study. Respir Med. 2016;121:26–31. 27888988 10.1016/j.rmed.2016.10.014

[6] Ringshausen FC de Roux A Diel R Hohmann D Welte T Rademacher J. Bronchiectasis in Germany: a population-based estimation of disease prevalence. Eur Respir J. 2015;46:1805–7. 26293498 10.1183/13993003.00954-2015

[7] Lin JL Xu JF Qu JM. Bronchiectasis in China. Ann Am Thorac Soc. 2016;13:609–16. 26882271 10.1513/AnnalsATS.201511-740PS

[8] Lu D Li C Zhong Z. Changes in the airway microbiome in patients with bronchiectasis. Medicine (Baltim). 2023;102:e36519. 10.1097/MD.0000000000036519 PMC10727580 38115299

[9] Duan JL Li CY Jiang Y. Microbiological characteristics of the lower airway in adults with bronchiectasis: a prospective cohort study. Respir Res. 2024;25:283. 39020401 10.1186/s12931-024-02903-1 PMC11253380

[10] Huffnagle GB Dickson RP Lukacs NW. The respiratory tract microbiome and lung inflammation: a two-way street. Mucosal Immunol. 2017;10:299–306. 27966551 10.1038/mi.2016.108 PMC5765541

[11] Qian YY Wang HY Zhou Y. Improving pulmonary infection diagnosis with metagenomic next generation sequencing. Front Cell Infect Microbiol. 2021;10:567615. 33585263 10.3389/fcimb.2020.567615 PMC7874146

[12] Loeffelholz M Chonmaitree T. Advances in diagnosis of respiratory virus infections. Int J Microbiol. 2010;2010:126049. 20981303 10.1155/2010/126049 PMC2958490

[13] Tuon FF. A systematic literature review on the diagnosis of invasive aspergillosis using polymerase chain reaction (PCR) from bronchoalveolar lavage clinical samples. Rev Iberoam Micol. 2007;24:89–94. 17604424

[14] Miao Q Ma Y Wang Q. Microbiological diagnostic performance of metagenomic next-generation sequencing when applied to clinical practice. Clin Infect Dis. 2018;67(suppl_2):S231–40. 30423048 10.1093/cid/ciy693

[15] Niu S Liu D Yang Y Zhao L. Clinical utility of metagenomic next-generation sequencing in the diagnosis of invasive pulmonary aspergillosis in acute exacerbation of chronic obstructive pulmonary disease patients in the intensive care unit. Front Cell Infect Microbiol. 2024;14:1397733. 39071167 10.3389/fcimb.2024.1397733 PMC11272591

[16] Zhang X Chen H Lin Y. Diagnosis of non-tuberculous mycobacterial pulmonary disease by metagenomic next-generation sequencing on bronchoalveolar lavage fluid. Infect Drug Resist. 2023;16:4137–45. 37396070 10.2147/IDR.S417088 PMC10312351

[17] Baughman RP. Technical aspects of bronchoalveolar lavage: recommendations for a standard procedure. Semin Respir Crit Care Med. 2007;28:475–85. 17975775 10.1055/s-2007-991520

[18] Jeon YJ Zhou Y Li Y. The feasibility study of non-invasive fetal trisomy 18 and 21 detection with semiconductor sequencing platform. PLoS One. 2014;9:e110240. 25329639 10.1371/journal.pone.0110240 PMC4203771

[19] Maran MIJ Davis G DJ. Benefits of merging paired-end reads before pre- processing environmental metagenomics data. Mar Genomics. 2022;61:100914. 34864203 10.1016/j.margen.2021.100914

[20] Fang X Mei Q Fan X. Diagnostic value of metagenomic next-generation sequencing for the detection of pathogens in bronchoalveolar lavage fluid in ventilator-associated pneumonia patients. Front Microbiol. 2020;11:599756. 33335520 10.3389/fmicb.2020.599756 PMC7736608

[21] Jin X Li J Shao M. Improving suspected pulmonary infection diagnosis by bronchoalveolar lavage fluid metagenomic next-generation sequencing: a multicenter retrospective study. Microbiol Spectr. 2022;10:e0247321. 35943274 10.1128/spectrum.02473-21 PMC9431624

[22] Hill AT Sullivan AL Chalmers JD. British thoracic society guideline for bronchiectasis in adults. BMJ Open Respir Res. 2018;5:e000348. 10.1136/bmjresp-2018-000348 PMC6326298 30687502

[23] Huang J Jiang E Yang D. Metagenomic next-generation sequencing versus traditional pathogen detection in the diagnosis of peripheral pulmonary infectious lesions. Infect Drug Resist. 2020;13:567–76. 32110067 10.2147/IDR.S235182 PMC7036976

[24] Zou X Tang G Zhao X. Simultaneous virus identification and characterization of severe unexplained pneumonia cases using a metagenomics sequencing technique. Sci China Life Sci. 2017;60:279–86. 27921234 10.1007/s11427-016-0244-8 PMC7088591

[25] Shen D Lv X Zhang H. Association between clinical characteristics and microbiota in bronchiectasis patients based on metagenomic next-generation sequencing technology. Pol J Microbiol. 2024;73:59–68. 38437464 10.33073/pjm-2024-007 PMC10911701

[26] Finch S McDonnell MJ Abo-Leyah H Aliberti S Chalmers JD. A Comprehensive analysis of the impact of pseudomonas aeruginosa colonization on prognosis in adult bronchiectasis. Ann Am Thorac Soc. 2015;12:1602–11. 26356317 10.1513/AnnalsATS.201506-333OC

[27] Dicker AJ Lonergan M Keir HR. The sputum microbiome and clinical outcomes in patients with bronchiectasis: a prospective observational study. Lancet Respir Med. 2021;9:885–96. 33961805 10.1016/S2213-2600(20)30557-9

[28] Guan WJ Gao YH Xu G. Sputum bacteriology in steady-state bronchiectasis in Guangzhou, China. Int J Tuberc Lung Dis. 2015;19:610–9. 25868032 10.5588/ijtld.14.0613

[29] Prevots DR Marshall JE Wagner D Morimoto K. Global epidemiology of nontuberculous mycobacterial pulmonary disease: a review. Clin Chest Med. 2023;44:675–721. 37890910 10.1016/j.ccm.2023.08.012 PMC10625169

[30] Morimoto K Iwai K Yoshiyama T. Epidemiological characteristics of nontuberculous mycobacteriosis and bronchiectasis: comparative study using national mortality statistics from 1970 to 2015 in Japan. ERJ Open Res. 2023;9:00424–2022. 36814552 10.1183/23120541.00424-2022 PMC9940714

[31] Mirsaeidi M Hadid W Ericsoussi B Rodgers D Sadikot RT. Non-tuberculous mycobacterial disease is common in patients with non-cystic fibrosis bronchiectasis. Int J Infect Dis. 2013;17:e1000–4. 23683809 10.1016/j.ijid.2013.03.018 PMC4472485

[32] Aksamit TR O’Donnell AE Barker A. Adult patients with bronchiectasis: a first look at the us bronchiectasis research registry. Chest. 2017;151:982–92. 27889361 10.1016/j.chest.2016.10.055 PMC6026266

[33] Máiz L Vendrell M Olveira C Girón R Nieto R Martínez-García MA. Prevalence and factors associated with isolation of Aspergillus and Candida from sputum in patients with non-cystic fibrosis bronchiectasis. Respiration. 2015;89:396–403. 25924628 10.1159/000381289

[34] Poh TY Tiew PY Lim AYH. Increased chitotriosidase is associated with aspergillus and frequent exacerbations in South-East Asian patients with bronchiectasis. Chest. 2020;158:512–22. 32184111 10.1016/j.chest.2020.02.048

[35] Gao YH Guan WJ Xu G. The role of viral infection in pulmonary exacerbations of bronchiectasis in adults: a prospective study. Chest. 2015;147:1635–43. 25412225 10.1378/chest.14-1961 PMC7094490

[36] Hu C Huang L Cai M Wang W Shi X Chen W. Characterization of non-tuberculous mycobacterial pulmonary disease in Nanjing district of China. BMC Infect Dis. 2019;19:764. 31477038 10.1186/s12879-019-4412-6 PMC6719376

